# A cost-effective 3D-printed cement spacer reconstruction in a rare case of calcaneal Ewing sarcoma: a case report and surgical technique

**DOI:** 10.3389/fsurg.2026.1864578

**Published:** 2026-07-06

**Authors:** Abdulrahman Alaseem, Fahad Alshayhan, Mansor Aldaijy, Ziyad Alsuwailem, Rayan Alfayez, Waleed Albishi, Hisham Alsanawi, Ibrahim Alshaygy

**Affiliations:** 1 Department of Orthopedic Surgery, College of Medicine, King Saud University, Riyadh, Saudi Arabia; 2 College of Medicine, King Saud University, Riyadh, Saudi Arabia

**Keywords:** 3D printing, calcanectomy, calcaneus, cement spacer, Ewing sarcoma, limb salvage

## Abstract

**Background:**

Ewing sarcoma (ES) accounts for 15% of primary bone tumors and is the second most common type seen in children and young adults between ages 5 and 20, with a male-to-female ratio of 1.5 to 1. Around one-third of patients have metastatic disease at diagnosis, while involvement of the foot is rare, accounting for only 5% of cases. For patients without metastases, treatment typically includes neoadjuvant chemotherapy, limb salvage surgery when possible, and adjuvant chemotherapy. Recent advances in 3D printing enable customized, patient-specific reconstruction in complex anatomical sites, such as the calcaneus.

**Case presentation:**

A 19-year-old female, previously healthy, presented with progressive left heel pain for 1 year without constitutional symptoms or trauma. Clinical observation revealed a swollen heel with intact overlying skin, no deformity, and no limitation of range of motion or neurovascular deficits. Radiologic local and systemic staging showed a nonmetastatic, aggressive, lytic, ill-defined lesion in the left calcaneus on x-ray, with MRI demonstrating a heterogeneously enhancing calcaneal tumor with soft-tissue extension into the heel fat pad and sinus tarsi. Histopathology of the image-guided true-cut biopsy specimen confirmed the diagnosis of Ewing sarcoma and primitive neuroectodermal tumor with EWSR1-FLI1 translocation. After multidisciplinary tumor board discussion, the patient received neoadjuvant chemotherapy with alternating vincristine, doxorubicin, cyclophosphamide, ifosfamide, and etoposide, resulting in tumor regression and good response on restaging. She then underwent limb salvage total calcanectomy. Reconstruction involved a custom-made, 3D-printed mold to fabricate an antibiotic-impregnated polymethylmethacrylate (PMMA) cement spacer, secured with two cannulated screws to the talus and Achilles tendon reattachment. After initial closure, the patient developed wound dehiscence, requiring a free radial forearm flap. She resumed adjuvant chemotherapy. At 1-year follow-up, the patient ambulated with a cane and a Toronto Extremity Salvage Score of 62.5%. By 2.5 years, she developed a deep surgical site infection (Enterococcus faecalis), managed by multiple irrigations, debridement, and implant removal. The infection resolved, the wound healed, and there were no signs of recurrence or metastasis.

**Conclusions:**

This case underscores the feasibility of limb salvage surgery in calcaneal primary malignant neoplasm, using versatile reconstructive options including custom 3D-printed prosthetic implants, cement spacers, and total calcanectomy without reconstruction.

## 1 Introduction

Ewing sarcoma (ES) is a malignant bone tumor representing 15% among all primary bone tumors and the 2nd most common malignant tumor among children and young adults (1). The predominantly affected age group is 5-20 years, with a male-to-female ratio of 1.5:1 (1–3). Approximately one-third of patients present with distant metastasis, involving the lungs and bone marrow, which are typical metastatic sites (2). The diaphysis of long bones is the most involved site and, to a lesser extent, the pelvis, ribs, and vertebrae (4). Nevertheless, ES can occur anywhere in the skeleton, although hand and foot involvement is rare, accounting for only 5% (4). Typical presentation of ES is non-mechanical pain, occasionally associated with constitutional symptoms such as fever, malaise, and weight loss; laboratory investigations may reveal leukocytosis or elevated inflammatory markers (5).

Non-metastatic ES is managed with neoadjuvant chemotherapy, limb-salvage wide resection, and adjuvant chemotherapy, while radiation therapy is indicated in certain circumstances. Surgical intervention depends on lesion extent, location, neurovascular involvement, functional outcome, and the availability of reconstruction resources (6).

3D printing has advanced reconstructive surgery by providing patient-specific 3D-printed plates to correct deformity and malunion, resulting in improved functional outcomes and more accurate alignment (7). Furthermore, cutting guides have been pivotal in enhancing preoperative templating and precise bone tumor resections, thus enhancing patient outcomes (8). 3D-printed prostheses have shown promising effectiveness, enabling early rehabilitation in oncological cases (9).

In this case report, a rare case of Ewing’s sarcoma of the calcaneum is described, treated with total calcanectomy followed by reconstruction with a cost-effective 3D-printed anatomical cement spacer mold.

## 2 Case presentation

### 2.1 History and physical examination

We report the case of a 19-year-old, previously healthy female who presented with persistent left ankle and heel pain for one year before presentation. With time, she started to notice progressive lateral swelling and pain with associated subjective fever, but no other constitutional or musculoskeletal symptoms. She denied any history of trauma, twisting, or sports injury.

Examination revealed an antalgic gait, swelling of the left lateral heel, no skin changes, tenderness to palpation, and a full, painless range of motion of the ankle and subtalar joints, with intact neurovascular examination. Laboratory workup revealed normal values except for mild leukocytosis (11.9), elevated erythrocyte sedimentation rate (41), and elevated C-reactive protein (14.9). Plain radiographs demonstrated multiple patches of radiolucency representing a lytic, ill-defined lesion in the calcaneus with no pathological fracture (Figure 1). Magnetic Resonance Imaging (MRI) showed an expansile, heterogeneous lesion with soft-tissue extension to the fat pad & sinus tarsi (Figure 2). Computed tomography (CT) scans of the chest, abdomen, and pelvis, along with a Fluorine-18 Fluorodeoxyglucose Positron Emission Tomography Computed Tomography (18F-FDG PET-CT) scan, showed no evidence of distant metastasis.

**Figure 1:**
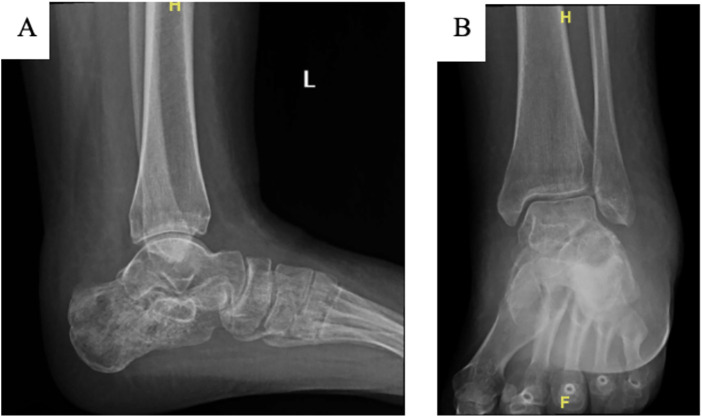
Lateral **(A)** and anteroposterior (AP) **(B)** radiographs of the left calcaneus demonstrating multiple patchy radiolucent lesions involving the calcaneus.

**Figure 2:**
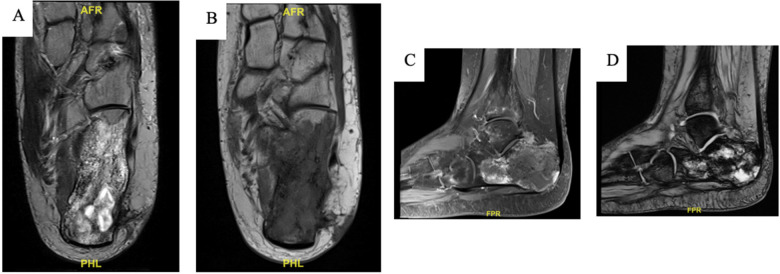
**(A)** Axial MRI-T2 and **(B)** axial MRI-T1 showing an expansile left calcaneal lesion with soft tissue extension to the fat pad. **(C)** Sagittal MRI-T1 with fat suppression with gadolinium contrast, and **(D)** sagittal MRI-T2 showing heterogeneous enhancement.

Upon Histopathological assessment, bone biopsy confirmed ES, revealing malignant round cells and an EWSR1-FLI1 gene fusion. Immunohistochemistry revealed strong positivity for CD99, and focal, weak expression of FLI1, CD117, and S100. while TdT, CD45, MPO, desmin, and CK-PAN were negative.

A multidisciplinary sarcoma tumor board planned neoadjuvant chemotherapy with Vincristine, Doxorubicin, and Cyclophosphamide, alternating with Ifosfamide and Etoposide (VDC/IE) every 3 weeks for 6 cycles. Given the patient’s young age, favorable prognosis, and nonmetastatic disease, amputation was excluded. A decision was made for total calcanectomy, followed by reconstruction with a 3D-printed cement spacer, given the lack of availability of a custom-made endoprosthesis due to logistical constraints during the COVID-19 pandemic.

### 2.2 Pre-operative planning

A 3D-printed calcaneal mold was created to ensure anatomical accuracy. The template size was measured from a mirrored CT Image of the contralateral calcaneus using image segmentation and mirror transformation. The mold was fabricated using the Anycubic Photon Mono X Printer (also known as Elegoo Mars 3) and the CHITUBOX Basic v1.9.5 and CHITUBOX Pro v2.0 software, with a non-medical, water-washable resin, and was sterilized with low-temperature hydrogen peroxide plasma prior to use. A sterile antibiotic-impregnated polymethyl methacrylate was injected into the mold to form the implant cement spacer. This option was a cost-effective, time-saving approach to preserving the limb and function.

## 3 Surgical technique

### 3.1 Tumor resection

A multidisciplinary team, including orthopedic oncology, foot and ankle surgery, and plastic surgery, performed the procedure under general anesthesia and prophylactic antibiotic administration. The patient was placed in a prone position, with A tourniquet applied to the proximal thigh. A Cincinnati approach was utilized, with the biopsy tract via an elliptical incision around it. Initially, the posteromedial neurovascular bundle was explored and secured. Then attention shifted to the lateral side, where the peroneal tendons were identified and isolated. Subsequently, the heel fat pad was raised, and the Achilles tendon was detached. The calcaneus was meticulously detached from the subtalar, calcaneocuboid, and navicular articulations. The surrounding ligamentous and soft-tissue attachments were dissected until the calcaneus was sequentially separated from the surrounding tissues, and a complete, wide-margin resection was achieved (Figure 3). The specimen was then sent for histopathologic evaluation, which confirmed gross negative margins. Copious irrigation with normal saline and iodine solution was performed. Subsequently, a 3D printed calcaneus cement spacer mold was assembled on the back table (Figure 4).

**Figure 3:**
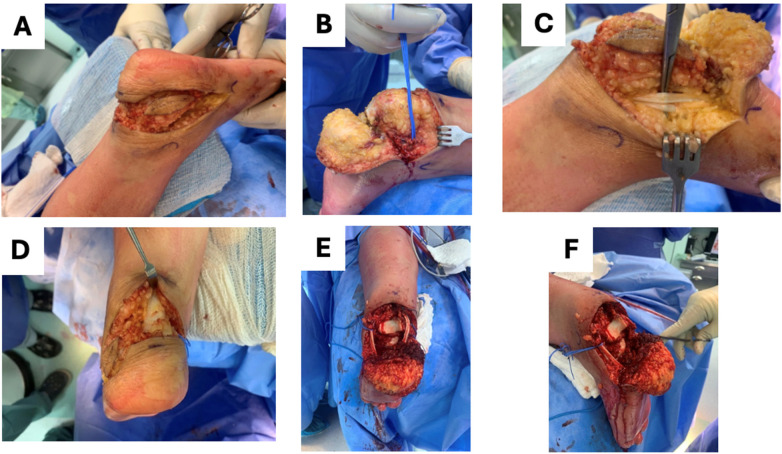
**(A)** Lateral limb of the cincinnati approach. **(B)** Secured posteromedial neurovascular bundle. **(C)** The peroneal tendons were isolated laterally. **(D)** Showing the Achilles tendon, which was later detached. **(E,F)** Wide-margin calcaneal excision, with detached Achilles tendon, isolated medial neurovascular bundle, and exposed talus.

**Figure 4:**
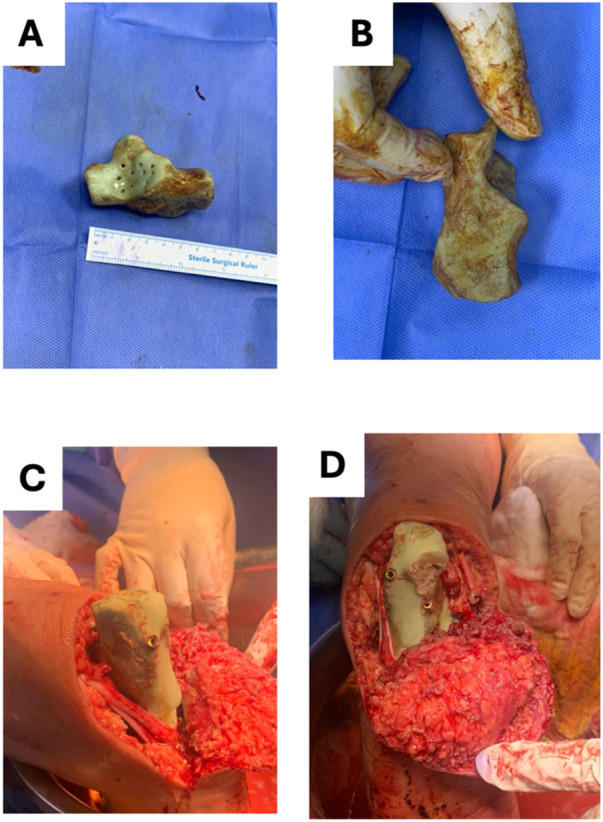
**(A,B)** 3D printed cement spacer of the calcaneus. **(C,D)** Lateral & Posterior picture showing the cement spacer fixation with two canulated screws to the talus.

### 3.2 Calcaneal reconstruction

The custom PMMA calcaneal cement spacer was inserted into the defect and fixed to the talus with two cannulated screws under fluoroscopic guidance (Figures 4, 5). To prevent weakening the calcaneal cement construct, we avoided drilling additional tunnels for Achilles tendon reconstruction. The Achilles tendon was attached to the talus using a Krakow technique with nonabsorbable number 5 Ethibond sutures. Fixation was augmented with suture anchors placed in the talus for secure tendon reattachment. While the posterior aspect of the plantar fascia was resected as part of the soft-tissue margin of the tumor, the remaining part was reattached to the deep subcutaneous tissue of the heel fat pad. After closure, the ankle was immobilized in plantar flexion with a cast.

**Figure 5:**
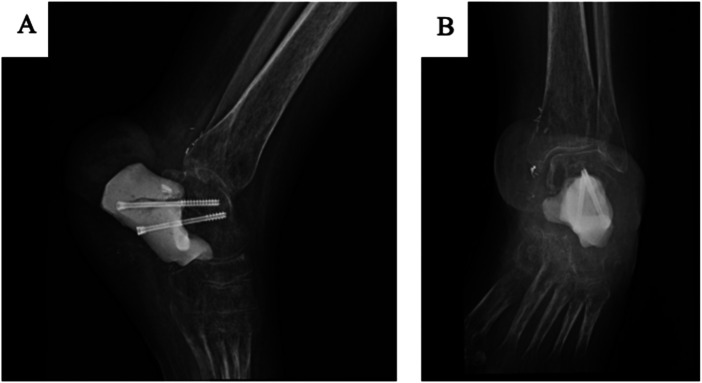
Demonstrating in pictures **(A,B)**, lateral and AP plain radiographs postoperative showing calcaneal cement spacer fixed to talus with two screws.

Two weeks postoperatively, a wound dehiscence measuring 19 × 11 cm was identified (Figure 6). Skin debridement was performed for the necrotic tissue. Plastic surgery was involved, and a decision was made to use a free pedicled radial forearm flap to cover the skin defect and improve wound healing. Upon subsequent follow-up, the flap was viable and healed (Figure 7). After wound healing, adjuvant chemotherapy was resumed with (VDC/IE) and Granulocyte-Colony Stimulating Factor (G-CSF) every 3 weeks for 10 cycles.

**Figure 6:**
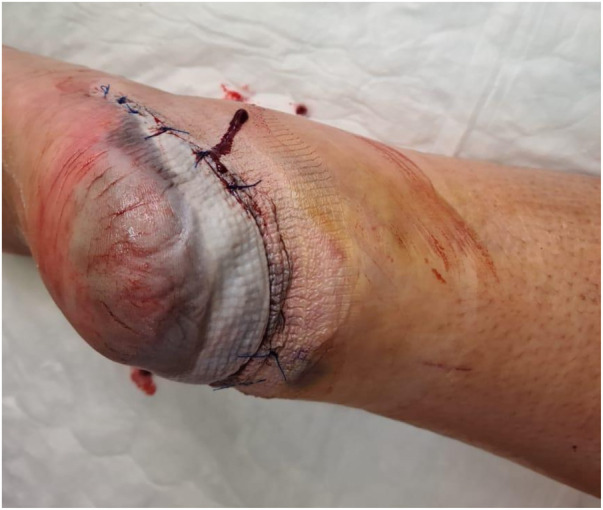
Two-week post-operative wound, with dehiscence with a 19 × 11 cm necrotic patch.

**Figure 7:**
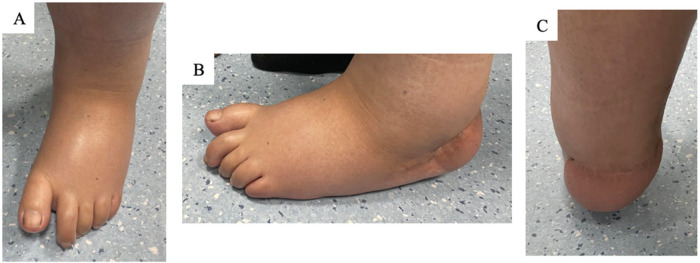
**(A–C)** demonstrating the anteroposterior, lateral, and posterior views of the foot at one-year post-operative visit in the clinic, showing healed scar.

### 3.3 Long-term outcome

One year after the surgery, the patient exhibited satisfactory functional recovery, pain resolution, wound healing, and ambulation with a cane, with good plantarflexion power at 30 degrees and limited dorsiflexion, and a Toronto Extremity Salvage Score (TESS) of 62.5% (Figure 7). Unfortunately, 2.5 years after the surgery, the patient presented to the Emergency department with a 2-day history of pain in the left foot with erythema, hotness, tenderness, and yellowish discharge from a sinus tract on the plantar aspect of the left heel with associated fever. Lab workup showed elevated Leukocyte, ESR, and CRP levels. She was admitted, started on antibiotics, and cultures were positive for Enterococcus faecalis, consistent with deep periprosthetic infection. Radiographic evaluation revealed no mechanical implant failure or loosening (Figure 8).

**Figure 8:**
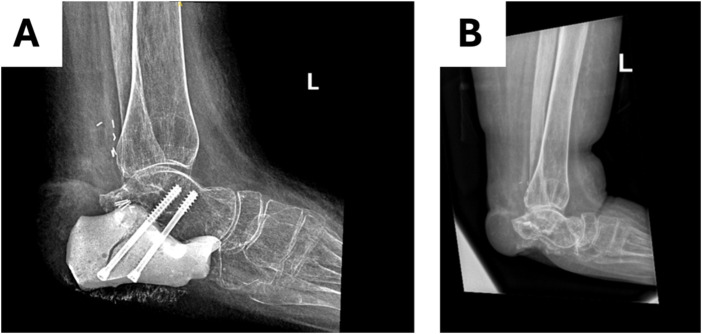
Demonstrating left foot lateral plain radiography **(A)** cement spacer with intact structural integrity with no fracture, fragmentation, or loosening. **(B)** Post-operative radiography after implant removal.

She underwent multiple surgical irrigations and debridements, then removal of the implant. Upon gross assessment, the cement spacer’s structural integrity was intact, with no evidence of fracture, fragmentation, or loosening. However, purulent discharge was observed, indicating the need for implant removal (Figure 8).

Following treatment, the infection resolved, and the sinus wound healed. She underwent intensive physical and occupational therapy. At the last follow-up, 3 years after the Implant removal, the functional assessment using the TESS was 54.5%. The patient was satisfied with her functional outcome. She was able to ambulate on her talus and the rest of her foot with rocker-bottom shoes and a cane for support. Imaging showed no signs of local recurrence or metastasis. Given her history of deep periprosthetic infection, further secondary prosthetic reconstruction was not pursued.

## 4 Discussion

Calcaneal ES involvement is exceedingly rare, requiring a high index of suspicion and thorough clinical, radiological, and histopathological workup (4). The multidisciplinary management includes neoadjuvant chemotherapy, adjuvant chemotherapy, and surgical intervention (6). When limb salvage is selected for local tumor control, reconstruction options include biological techniques, prosthetic replacement, or resection without reconstruction. These methods typically result in higher patient satisfaction and functional outcomes that are comparable to those achieved with amputation (6, 10, 11). Nevertheless, the anatomical complexity of foot and ankle tumors, weight-bearing role, limited availability of endoprosthesis, and reliable limb-preserving reconstructive techniques frequently lead surgeons to select amputation as the preferred procedure (2).

Among limb-salvage techniques, Biologic reconstruction after total calcanectomy, using an allograft rather than an iliac crest autograft, has yielded good functional outcomes; however, it carries a greater risk of infection and allograft reabsorption (6). Alternatively, calcaneectomy without reconstruction has been described in a case series in which patients underwent excision arthroplasty of the subtalar joint, followed by reattachment of the Achilles tendon to the talus. The reported mean functional score on the TESS was 88.5%. However, the patients developed a two-centimeter limb-length discrepancy, and ambulation was assisted with a customized rocker-bottom shoe (11). Our patient underwent a similar procedure with complications, including infection and repeated debridement, as one of the reported cases with an early TESS of 58.5% (11). This modest functional outcome may reflect the cumulative burden of heel fat pad excision, infection, prolonged chemotherapy, and delayed rehabilitation.

Recent Advances in 3D printing technology have transformed orthopedic surgical procedures, particularly in complex skeletal reconstruction, by enabling patient-specific implants that optimize load distribution and provide reproducible sites for tendon or ligament reattachment (9, 12). Evidence from case reports and series indicates that 3D-printed talus-calcaneus prostheses restore limb length and alignment, achieving favorable functional outcomes (e.g., MSTS scores up to 27/30; AOFAS scores up to 92/100), satisfactory range of motion, and high patient satisfaction at short-term follow-up (9, 13). However, it remains essential to balance its mechanical benefits against cost, logistical constraints, infection risk, and long-term durability (14).

PMMA cement spacers have been described as both temporary and definitive hindfoot reconstructive options, providing immediate restoration of length and alignment, antibiotic elution, while maintaining low cost and intraoperative versatility (9, 13).

Compared to metal prostheses, PMMA spacers have weaker biomechanical properties, lack long-term durability, and are more prone to cement fracture and mechanical failure. The articulating surface of a PMMA cement spacer is relatively rough, which can lead to arthritic changes in adjacent joints. Moreover, the literature reports that porosity and elevated antibiotic loads significantly affect mechanical performance, with high antibiotic concentrations substantially reducing bending strength within several weeks, with fracture rates for articulating, weight-bearing spacers ranging from 2% to 24% (14). However, Reinforcement of PMMA with metal endoskeletons or hardware consistently improves flexural strength and residual stability during bending and cyclic loading, and porous design of the implant helps with the implant’s osteointegration (9, 15). While PMMA spacers alone exhibit inherent limitations in flexural strength and long-term fatigue performance under cyclic loading, the mechanical durability of the construct in this young patient was strategically optimized. By anchoring the anatomical spacer with two large-diameter cannulated screws rigidly fixed to the talus, we effectively transformed the PMMA into a reinforced composite structural block. This stabilization drastically improved structural stiffness and resistance to bending stress, allowing the construct to withstand high-impact weight distribution and cyclic physiological loading during early mobilization without fragmentation or component displacement. However, clinical studies assessing the long-term survival of 3D-printed PMMA constructs as permanent load-bearing implants remain scarce. In contrast, custom metal endoprostheses offer a smoother, structurally superior articulating surface with significantly better long-term fatigue life under continuous physiological stress.

When considering the cost and logistical constraints associated with 3D-printed metal endoprostheses, improvising a 3D-printed mold to create a PMMA anatomical spacer is a creative yet practical, cost-effective approach that allows surgeons to design patient-specific molds and spacers, thereby broadening limb salvage options, especially when allografts or custom prostheses are unavailable. The adaptability of in-house 3D printing was emphasized during the COVID-19 pandemic, when it was used to locally manufacture medical device components in response to global supply chain disruptions, underscoring its importance in resource-constrained, urgent situations (16).

Postoperatively, our patient unfortunately experienced early wound dehiscence and skin necrosis, which was managed with uneventful free flap reconstruction. Subsequently, another complication occurred two years later: a plantar heel sinus with associated deep infection. This was most likely attributable to progressive weight-bearing on the hindfoot in the setting of limited soft-tissue coverage, particularly following excision of the calcaneal fat pad due to tumor involvement. The infection was managed with IV antibiotics, serial irrigation, and debridement, followed by implant removal. Spacer utilization in foot and ankle oncology cases is associated with high infection rates, ranging from 20 to 40 percent, due to their weight-bearing nature, tissue loss, foreign-body exposure, and prior radiotherapy (17). When reconstruction fails, often due to infection or mechanical issues, patients may require excision arthroplasty or even amputation. While many can still walk with assistive devices, challenges like limb-length differences and altered gait are common. Custom prostheses usually offer a quicker return to mobility, whereas biological reconstructions can provide better long-term outcomes if successful, but involve longer, more complex recovery. Amputation remains an effective last resort, though it brings its own difficulties, such as chronic pain and the need for major life adjustments. Each approach comes with trade-offs that can significantly affect a patient’s quality of life.

## 5 Conclusions

This case highlights the complexity of managing Ewing’s sarcoma of the calcaneus, a rare clinical entity historically associated with poor outcomes in the past, due to its complex anatomy, biomechanics, and oncologic features. Effective management in this particular patient required complete calcanectomy, unique reconstruction with 3D-printed cement, and complex microsurgical reconstruction for tissue coverage. Even in the presence of extreme morbidity such as wound failure and late deep infection requiring cement removal, dedicated care enabled lasting limb salvage, locoregional disease control, and satisfactory functional outcomes with perserved quality of life.

## 6 Limitations

This report relies on a single patient, thereby limiting generalizability. Extensive studies on reconstruction techniques, such as 3D-printed spacers, and their oncological and functional durability have yet to be conducted.

## Data Availability

The original contributions presented in the study are included in the article/Supplementary Material, further inquiries can be directed to the corresponding author.
